# Screening for eating disorders in adolescents with chronic pain: the Eating Attitudes Test–16–Chronic Pain

**DOI:** 10.1186/s40337-024-01014-y

**Published:** 2024-05-10

**Authors:** Leslie Sim, Amy Fahrenkamp, Jennifer R. Geske, Jocelyn Lebow, Hope Thilges, Carol B. Peterson, Abigail Matthews, Cynthia Harbeck-Weber

**Affiliations:** 1 Department of Psychiatry and Psychology, Mayo Clinic College of Medicine and Science, 200 First Street SW, Rochester, MN 55905 USA; 2 Pain, Palliative Care, and Integrative Medicine Department, Children’s Hospitals and Clinics of Minnesota, Minneapolis, MN USA; 3 Department of Quantitative Health Sciences, Mayo Clinic College of Medicine and Science, Rochester, MN USA; 4 Department of Pediatric and Adolescent Medicine, Mayo Clinic College of Medicine and Science, Rochester, MN USA; 5 Department of Psychology, University of Nebraska-Lincoln, Lincoln, NE USA; 6 Department of Psychiatry and Behavioral Sciences, University of Minnesota Medical School, Minneapolis, MN USA

**Keywords:** Eating disorders, Chronic pain, Adolescents, Factor analysis

## Abstract

**Background:**

Few measures have been validated to screen for eating disorders (ED) in youth with chronic pain. We conducted confirmatory (CFA) of two established factor structures of the Eating Attitudes Test-26 (EAT-26) in a sample of youth with chronic pain attending an intensive interdisciplinary pain treatment (IIPT) program and examined the validity of the best-fitting model in predicting ED diagnoses in this sample.

**Methods:**

Participants were 880 adolescents (*M* age = 16.1, *SD* = 2.1) consecutively admitted into an IIPT program who completed the EAT-26 upon admission. CFA was conducted and in the case of inadequate fit, EFA was planned to identify alternative models. Factors of the best-fitting model were included in a logistic regression analysis to predict ED diagnoses.

**Results:**

The TLIs (0.70; 0.90), RMSEAs (0.09; 0.07) and CFIs (0.73; 0.92) suggested poor fit of one model and adequate of the second model. Goodness of fit indices from EFA (TLI:0.85, RMSEA:0.06) did not outperform the fit of the second CFA. As such, the second model was retained with the exception of one factor. The items loaded onto a 16-item, five factor model: *Fear of Getting Fat*, *Social Pressure to Gain Weight, Eating-Related Control, Eating-Related Guilt* and *Food Preoccupation*. Based on chart review, 19.1% of the participants were diagnosed with an eating disorder. Logistic regression analyses indicated the new 16-item measure and *Fear of Getting Fat*, significantly predicted an ED diagnosis that did not include avoidant restrictive food intake disorder (ARFID) and *Social Pressure to Gain Weight* significantly predicted a diagnosis of ARFID.

**Conclusions:**

An alternative 16-item, 5-factor structure of the EAT-26 should be considered in screening for EDs with youth with chronic pain.

## Pain English Summary

Youth with chronic pain are at risk for eating disorders, yet there are few validated measures to screen for these conditions in this population. The Eating Attitudes Test (EAT-26) is a widely used measure of eating disorders that has been evaluated on eating disorder and community samples. This study examined whether the EAT-26 and its widely used subscales are appropriate for use with adolescents with chronic pain. Results from confirmatory factor analyses indicate that a modification of a previous 6 factor model of the measure is relevant to the eating pathology of adolescents with chronic pain. This 16-item measure with 5 subscales (*Fear of Getting Fat*, *Social Pressure to Gain Weight, Eating-Related Control, Eating-Related Guilt*, and *Food Preoccupation)* better fits this population. The shorter measure, *EAT-16-Chronic Pain*, and new scales were associated with eating disorder diagnoses in the sample of youth with chronic pain, suggesting their validity for use with this population.

## Background

Many adolescents with chronic pain (pain lasting longer than 3–6 months) identify eating as a central area of concern [1–5]. Adolescents with chronic pain commonly restrict their dietary intake in response to pain, nausea, poor appetite, disrupted sleep-wake cycles, medication side effects and elimination diets [3, 5–8]. Although dietary alterations may achieve a temporary reduction in physical symptoms, if these behaviors persist, such dietary restriction may increase the risk for the development of an eating disorder [1, 3, 7, 9].

In fact, research highlights that a subset of adolescents with pain-related restrictive eating go on to develop drive for thinness, fear of weight gain, and purposeful efforts to control weight and shape [5]. This shift in motivation for restrictive eating from efforts to manage pain to controlling weight and shape may develop from starvation-related changes to the brain, fasting-related reduction in anxiety, as well as social reinforcement for weight loss [9–11]. In addition to risk for the development of eating disorders involving weight or shape concerns, youth with chronic pain are also at risk for avoidant restrictive food intake disorder (ARFID). Similar to ARFID, adolescents with chronic pain frequently restrict their diet in the context of aversive gastrointestinal symptoms, sensory sensitivities, and/or low appetite, dietary restriction that poses consequences to their health and social functioning [12].

In spite of findings indicating a higher prevalence of eating disorders across various chronic pain conditions including migraine headache, functional gastrointestinal disorders, fibromyalgia, and chronic facial pain [13–18], research on the overlap between eating disorders and chronic pain has been limited. One of the few studies to examine this relationship found adolescents with chronic pain and comorbid eating disorders take longer to be identified and referred for eating disorder treatment than a matched sample of adolescents without chronic pain [5]. This delay may stem in part from the lack of appropriate measures to screen for eating disorders. The absence of eating disorder screening measures for this population also limits our understanding of how to best prevent and treat eating disorders in these youth [19, 20].

The Eating Attitudes Test − 26^21^ is one of the most widely used instruments to screen for disordered eating attitudes and behaviors [22]. It has been examined in a range of populations including adults and adolescents with eating disorders, athletes, community samples, as well as has been examined across many cultures [22]. However, it has not been evaluated in adolescents with chronic pain or those with ARFID. Given data on adolescent norms [23], as well as the short administration time and straightforward scoring, this measure has potential for screening for eating disorders in a chronic pain population.

The original factor structure of the EAT-26, initially derived from an all-female sample and later replicated with adolescent school-aged girls, includes *Dieting* (i.e., food avoidance and drive for thinness), *Bulimia* (i.e., cognitions about food and binge-eating/purging items), and *Oral Control* (i.e., social pressures to gain weight and self-control over eating) [24]. However, factor analytic studies with adolescents have largely failed to replicate Garner et al.’s 3 factor solution, particularly among non-clinical samples [24–28]. In mixed gender, community samples, an alternative factor structure has been identified and replicated for the EAT-26 [27, 28]. Given that at least one third of youth with chronic pain identify as male, this model may have more relevance to screening for eating disorders in youth with chronic pain [29].

Given the need for eating disorder screening in patients with chronic pain, the primary goal of the study was to conduct a confirmatory factor analysis on two established models in a sample of adolescents attending an intensive, interdisciplinary pain treatment (IIPT) program. These models include Garner’s original factor structure of the EAT-26 and an alternative 6-factor model (i.e., Fear of Getting Fat, Social Pressure to Gain Weight, Vomiting-Purging Behavior, Eating Related Control, Food Preoccupation, Eating Related Guilt) identified by Maiano’s (2016) [27] factor analysis and replicated by McEnery (2016) [28]. Given the mixed gender sample of youth who attend the IIPT, we hypothesized that the Maiano model would yield a better fit for the data.

In the case of less than adequate fit, we planned to conduct exploratory factor analysis to identify an alternative model that better fit the data. A final aim of this study was to examine whether the factors of the best-fitting model predict eating disorder diagnoses based on chart review. In the process of the chart review, we examined the prevalence of adolescents who scored above clinical cutoffs on the EAT-26, as well as of those who received an eating disorder diagnosis. We hypothesized that the prevalence of eating disorders, as well as ARFID would be higher than the general population.

## Materials and methods

### Participants

Participants were 880 adolescents (mean age = 16.1, *SD* = 2.1; 72.4% female) consecutively enrolled in a pediatric IIPT program at a tertiary medical center in the Midwestern United States between 2013 and 2018. This IIPT program serves patients from across the United States with high impact non-cancer chronic pain and comorbid conditions. Participants identified as predominantly White (*n* = 794; 90.2%) and resided in the United States, the Virgin Islands, and Canada. At the time of their participation, the majority were in high school (74.7%). See Table 1 for detailed demographic characteristics of participants.

**Table 1:** Participant characteristics

Participant characteristic	Original Sample (*n* = 880)	
	Mean	SD
Age	16.1	2.1
Body Mass Index (kg/m2)	24.2	6.5
Body Mass Index Percentile	63.8	30.3
Duration of Pain (years)	3.6	3.3
Eating Attitudes Test − 26	11.1	9.5
**Participant characteristic**	**N**	**%**
Female	637	72.4
Race		
White	794	90.2
Black	12	1.4
Asian	18	2.1
Indigenous American	9	1.0
Native Hawaiian/Pac. Islander	1	0.1
Multiracial	38	4.3
Other	8	0.9
Primary Pain Diagnosis		
Headache/migraine	369	41.9
Abdominal pain/Gastrointestinal symptoms	200	22.7
Musculoskeletal pain	217	24.7
Other	94	10.7
EAT-26 score *≥* 20	164	18.6
Eating Disorder Evaluation Status	200	22.7
Any Eating Disorder	168	19.1
AN, BN, BED, or OSFED*	62	7.1
AN	11	1.3
BN	8	0.9
BED	7	0.8
OSFED-Restrict	26	3.0
OSFED-Binge	9	1.0
Avoidant Restrictive Food Intake Disorder	107	12.2

Note. *Indicates *n* = 1 Unspecified eating disorder. Other primary pain diagnosis = any pain diagnosis that could not be categorized into the other 3 categories. AN = Anorexia Nervosa. BN = Bulimia Nervosa, BED = Binge Eating Disorder, OSFED-Restrict = Other Specified Feeding and Eating Disorder primarily involving restrictive eating, OSFED-Binge = Other Specified Feeding and Eating Disorder primarily involving binge eating/and or purging behavior

### Measures

#### Eating attitudes test-26 (EAT-26)

The Eating Attitudes Test – 26 (EAT-26; Garner et al., 1982) [21] is a 26-item, self-report measure used to assess disordered eating attitudes and behavior. Items are rated on a six-point scale from “never” to “always.” Scores on each item are recoded such that a score of 0 is assigned to the first 3 scores in the least symptomatic direction and the 3 scores in the symptomatic direction are weighted 1,2,3, respectively. The recoded items are summed with a clinical screening cutoff of 20 or higher indicating significant concern for a potential eating disorder. The EAT demonstrates good sensitivity and specificity for identifying eating disorders in community and clinical samples [22].

### Procedure

As part of standard IIPT admission procedures, participants in both samples completed the EAT-26 along with other intake questionnaires at the time of their admission. The IIPT is a 3-week, intensive program for adolescents and young adults struggling with chronic pain that takes place from 8am to 4pm each weekday. Participants engage in cognitive-behavior therapy, occupational therapy, physical therapy, recreation therapy and biofeedback in a group setting and receive individual and family interventions, as well as psychiatric and medical services. During the program, patients showing risk for psychiatric symptoms are referred for evaluation and brief interventions with a board-certified child and adolescent psychologist or psychology post-doctoral fellow under the supervision of a board-certified psychologist. In the current sample, most patients had an individual meeting with a psychologist, with 22.7% participating in a focused evaluation for an eating disorder.

Three independent coders extracted data from the adolescents’ electronic medical records from visits at Mayo Clinic beginning with participants’ initial visit to Mayo Clinicthrough all subsequent medical record documents including medical and psychiatric inpatient and outpatient visits. Coders included a doctoral level clinical psychologist, a postdoctoral clinical psychology fellow, and a bachelor’s level research assistant who underwent training by the study co-PIs (LAS and AF) to systematically review records for evidence of DSM-5 feeding and eating disorders criteria.

Coders extracted data from the nursing admission note which included systematically collected information regarding eating disorder history, dietary intake, eating behavior and weight, as well as the notes from the patients’ episode of care while they were in the program on: (1) primary pain diagnoses (diagnosed by a board-certified pediatric pain physician), (2) body mass index (BMI) percentile (adjusted for age and biological sex) at admission, (3) eating disorder history, and (4) formal eating disorder diagnoses in the medical record associated with eating disorder assessment. To confirm the eating disorder diagnoses listed in the medical record, coders extracted chart information related to DSM-5 diagnostic criteria for eating disorders including anorexia nervosa (AN), bulimia nervosa (BN), binge-eating disorder (BED), other specified feeding and eating disorder (OSFED) and avoidant restrictive food intake disorder (ARFID), and only included the chart diagnosis if it was consistent with diagnostic criteria. 20% of the records were extracted by an independent rater (LS) who reached 94.7% agreement (Kappa = 0.84). Disagreements were resolved by consensus. Adolescents’ primary pain diagnosis was determined by the first listed pain diagnosis diagnosed by a board-certified pain physician associated with the program. The Mayo Clinic Institutional Review Board approved study procedures for this study. Only records from patients and/or parents/guardians who had provided research authorization were included.

### Data analyses

Participant characteristics, eating disorder diagnoses, and scores on the EAT-26 were summarized with mean and standard deviation or N and percent (See Table 1). To examine the fit of Garner’s original 3-factor model [21] and Maiano’s 6-factor model [27] in our sample of youth with chronic pain, confirmatory factor analysis was conducted. To determine whether the models provided an acceptable fit to the data, model fit statistics, factor loadings, and modification indices were examined using the following criteria: (1) the Tucker-Lewis index (TLI) with values ideally 0.90 or greater, suggesting the model improves 90% relative to the null model; (2) The Root Mean Square Error Approximation (RMSEA) with values closer to 0 representing a better fit; (3) The Comparative Fit index (CFI) which is similar to the TLI with values ideally > 0.9; and the standardized root mean square residual (SRMR), defined as the difference between the residuals of the covariance matrix and the hypothesized models. The SRMR considers when items vary in range with optimal values < 0.08.

Exploratory factor analysis was conducted to examine whether a new factor structure better fit our sample using square multiple correlations as prior communality estimates with oblique rotation of the factors. The number of factors was ascertained using scree plots, proportion of common variance explained by the factors, parallel analysis, and interpretability of factors. Variables were considered to load on a factor if the factor loading was ≥ 0.40.

Finally, three BMI percentile-adjusted multivariable logistic regression models were conducted to examine the scores for all five factors of the best fitting model as predictors of: 1) a diagnosis of any eating disorder (i.e., AN, BN, BED, OSFED, ARFID; 2), a diagnosis of an eating disorder that does not include ARFID (i.e., AN, BN, BED, or OSFED); 3) or an ARFID diagnosis. Finally, multivariable models were run for each outcome with all factors included in the model, adjusted for BMI percentile. C-statistics measure binary models’ performance, with values greater than 0.7 indicating a good model fit and values greater than or equal to 0.8 indicating an excellent model fit. Data analyses were conducted using R (version 4.0.3) using the Psych and Lavaan package.

## Results

Confirmatory factor analysis of Garner’s original 3-factor model [21] with our sample of adolescents and young adults with chronic pain yielded a TLI (0.70), RMSEA (0.093, 90% CI: 0.090–0.096), and CFI (0.73) suggesting poor fit. Maiano’s 6-factor model [27] would not converge, due to too few subjects endorsing vomiting after eating for item 9. The reduced 5-factor model yielded a TLI (0.9), RMSEA (0.072, 90% CI: 0.066–0.78), and CFI (0.92) suggesting an improved fit; however, we hypothesized this could be further improved.

As such, exploratory common factor analysis was conducted to determine the most meaningful factor structure of the EAT-26 with our sample using square multiple correlations as prior communality estimates with oblique rotation of the factors. A new four-factor structure did not improve the fit with a TLI of 0.85 and RMSEA of 0.065 (90% CI: 0.061–0.069) in an EFA.

**Fig. 1:**
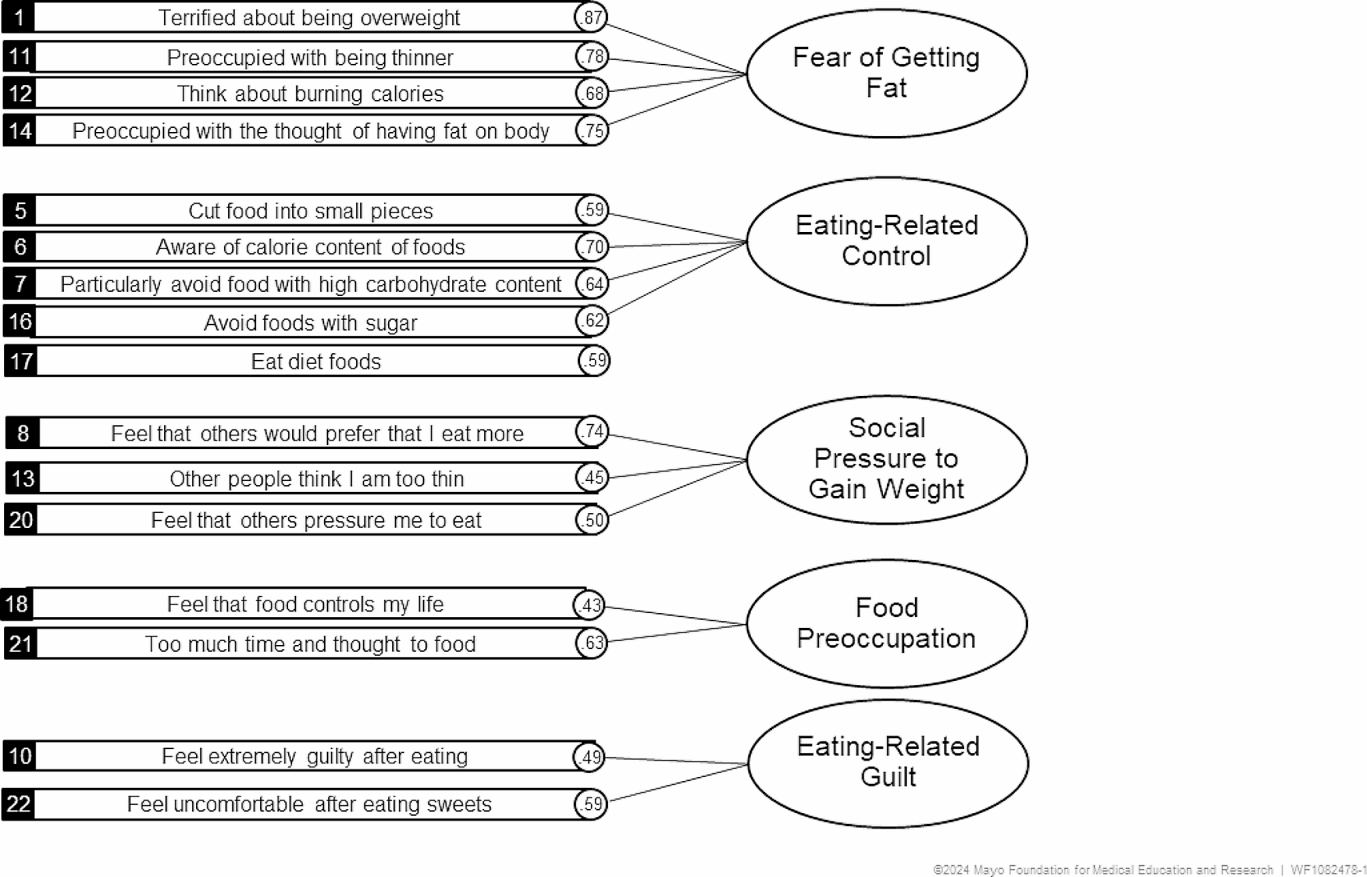
Factor structure of the Eating Attitudes Test− 16 - Chronic Pain

As shown in Fig. 1, Maiano’s 5-factor structure gives clearly interpretable factors previously documented as *Fear of Getting Fat*, (e.g., “I am preoccupied with a desire to be thinner,” “I am terrified of being overweight”), *Social Pressure to Gain Weight* (e.g., “I feel that others would prefer if I ate more,” “I feel that others pressure me to eat,”), *Eating-Related Control* (e.g., “I avoid foods with sugar,” “I eat diet foods”), *Food Preoccupation* (e.g., “I feel that food controls my life”) and *Eating-Related Guilt (e.g.,"I feel extremely guilty after eating")*. Cronbach’s Standardized alpha was 0.85 overall and ≥ 0.74 for each factor demonstrating good or better reliability. The hierarchical Omegas (ω_*h*_) similarly demonstrated sufficient (ω_*h*_ = 0.66 for Eating-Related Control; 0.67 for Eating-Related Guilt) and excellent reliability (ω_*h*_ = 0.88 for Fear of Getting Fat; ωh = 0.78 for Social Pressure to Gain Weight and Food Preoccupation).

Based on chart review, 19.1% of the sample of youth with chronic pain attending an intensive, interdisciplinary pain treatment program were diagnosed with an eating disorder. Of this sample, 7.1% received an eating disorder diagnosis other than ARFID (e.g., AN, BN, BED, OSFED) and 12.1% were diagnosed with ARFID.

In examining BMI percentile-adjusted logistic regression models with the five factor scores as predictors, the factor, *Social Pressure to Gain Weight*, significantly predicted higher odds of an ARFID diagnosis (OR = 1.5, *p* < .01) and any eating disorder (OR = 1.4, *p* < .05). The factor, *Fear of Gaining Weight*, was negatively associated with any eating disorder diagnosis (OR = 0.5, *p* < .05). The factor, *Eating-Related Guilt*, significantly predicted receiving an non-ARFID eating disorder diagnosis (OR = 4.5, *p* < .05) and any eating disorder diagnosis (OR = 3.3, *p < .05)*. *Food Preoccupation* and *Eating-Related Control* did not predict any of our eating disorder related outcomes. These models showed good predictive (diagnostic) accuracy with C-statistics ranging from 0.77 to 0.80. See Table 2 for BMI percentile-adjusted logistic regression models for each of the four factors.

**Table 2:** Odds ratios of EAT-16 - Chronic Pain factors predicting eating disorder diagnoses

Outcome	Predictor	OR (95% CI)	C-statistic	*p*-value
ARFID dx	BMI percentile	0.98 (0.97, 0.99)	0.80	< 0.0001
	Fear of weight gain	0.46 (0.17, 1.23)		0.1217
	Social pressure to gain weight	1.48 (1.13, 1.93)		0.0047
	Food preoccupation	1.34 (0.83, 2.16)		0.2332
	Eating-related guilt	1.43 (0.34, 5.92)		0.7259
	Eating-related control	0.79 (0.45, 1.42)		0.4351
Non-ARFID ED	BMI percentile	0.99 (0.98, 0.99)	0.80	0.0181
	Fear of weight gain	0.59 (0.25, 1.42)		0.2418
	Social pressure to gain weight	0.87 (0.61, 1.24)		0.4313
	Food preoccupation	0.85 (0.53, 1.35)		0.4857
	Eating-related guilt	4.47 (1.32, 15.13)		0.0160
	Eating-related control	1.10 (0.69, 1.75)		0.6958
Any ED	BMI percentile	0.98 (0.97, 0.99)	0.77	< 0.0001
	Fear of weight gain	0.47 (0.23, 0.95)		0.0358
	Social pressure to gain weight	1.27 (1.01, 1.59)		0.0427
	Food preoccupation	1.10 (0.76, 1.57)		0.6192
	Eating-related guilt	3.34 (1.22, 9.11)		0.0188
	Eating-related control	0.88 (0.59, 1.32)		0.5453

BMI percentile-adjusted logistic regression models with the 16-item model as a predictor of eating disorder diagnoses and evaluation are presented in Table 3. Higher EAT-16 total scores were significantly associated with increased risk of all eating disorder diagnoses categories with the exception of ARFID (*p* = .17). These models showed good predictive (diagnostic) accuracy with C-statistics ranging from 0.75 to 0.77.

**Table 3:** Odds ratios examining EAT-16 - Chronic Pain total predicting eating disorder evaluation and eating disorder diagnoses, adjusting for BMI

Outcome	Predictor	OR (95% CI)	C-statistic	*p*-value
ARFID dx	BMI percentile	0.97 (0.96, 0.98)	0.75	< 0.0001
	EAT-16 total	1.02 (0.99, 1.06)		0.17
Non-ARFID ED	BMI percentile	0.99 (0.98, 1.00)	0.77	0.074
	EAT-16 total	1.13 (1.09, 1.16)		< 0.0001
Any ED	BMI percentile	0.97 (0.97, 0.98)	0.75	< 0.0001
	EAT-16 total	1.09 (1.06, 1.12)		< 0.0001

## Discussion

Based on the need for a reliable and valid screening measure for this population, we conducted a factor analysis of the EAT-26 in a sample of adolescents with chronic pain attending an IIPT program. Results suggest that youth with chronic pain demonstrate variant eating pathology that is not adequately captured in Garner’s original 3-factor solution for the EAT-26. This is consistent with prior research that similarly failed to replicate Garner’s original factor structure in a general population of mixed-gender adolescents [27, 28]. However, our confirmatory and exploratory factor analysis also did not replicate a 6-factor solution of the EAT-26 from Maiano’s et al., that was identified using mixed gender adolescent samples [27, 28]. Instead, our best-fit factor analysis was a version of Maiano et al.’s confirmatory factor analysis that loaded onto five of their six unique factors. The sixth factor lacked the endorsement of vomiting indicated in items 9 and 26, and therefore was incalculable. The lack of improved exploratory factor analysis may be related to our sample of youth with chronic pain with a unique gender distribution compared to the Maino and McEnery samples EAT-26 (72% vs. 50–60% female, respectively) [27, 28].

Findings of confirmatory factor analysis suggest a new scale which we titled, EAT-16-Chronic Pain. This model is a slight modification to the Maiano structure using a 16-item model with five unique factors *(Fear of Getting Fat*, *Eating-Related Guilt, Eating-Related Control, Social Pressure to Gain Weight*, *Food Preoccupation)* which is a better fit for young patients with chronic pain. Supporting the convergent and discriminant validity of the unitary construct to evaluate non-ARFID eating disorders in these youth, higher EAT-16 -Chronic Pain total scores were significantly associated with increased risk of all eating disorder diagnoses categories with the exception of ARFID (*p* = .17).

Similarly, the factor *Fear of Getting Fat* was associated with an eating disorder diagnosis not including ARFID (i.e., AN, BN, BED, OSFED) but not an ARFID diagnosis. However, adolescents’ score on the factor, *Social Pressure to Gain Weight,* did significantly predict an ARFID diagnosis. Because adolescents with ARFID may perceive food avoidance as adaptive to minimize aversive experiences, they may not view their eating as problematic but may instead recognize others’ concerns about their dietary intake and weight. Since many adolescents with chronic pain eliminate specific foods from their diet that they believe exacerbate pain, it was surprising that the third factor, *Eating-Related Control*, did not predict an ARFID diagnosis. It is possible that the particular type of control over eating that this scale captures is more closely tied to achieving weight loss rather than symptom reduction.

### Clinical and research implications

Our finding that a high proportion (19.1%) of adolescents attending an IIPT for treatment of high-impact, non-malignant chronic pain qualified for an eating disorder diagnosis highlights the importance of screening for eating disorders in this population. As a screening for non-ARFID related eating disorders (i.e., AN, BN, BED, OSFED), an alternative 16-item measure along with Factor 1, *Fear of Getting Fat*, showed convergent and discriminant validity, as they predicted all eating disorders except for ARFID. Factor 2, *Social Pressure to Gain Weight*, a scale comprising items that highlights the perception of others’ concerns about the patients’ eating and weight, was the only factor associated with an ARFID diagnosis. Given that *Social Pressure to Gain Weight* was associated with ARFID, in addition to the use of ARFID-specific assessment measures, inquiry into patients’ perceptions of eating and weight related concerns from others may represent a fruitful avenue to screen for ARFID-related eating pathology in this population. Clinically, the findings of this study have implications for brief assessment using an abbreviated 16-item version of the measure which may be useful in busy medical settings when evaluating these youth.

Our chart review of a large sample of youth with chronic pain identified that 19.1% of this sample met criteria for any eating disorder, with 7.1% meeting the criteria for a non-ARFID eating disorder (i.e., AN, BN, BED, OSFED) and 12.2% meeting criteria for ARFID. The study also found that 16.1% of the sample scored above clinical cutoffs on the EAT-26. The fact that fewer patients scored above clinical cutoffs on the screening measure than received eating disorder diagnoses is consistent with the large proportion of patients with ARFID in this sample which is not adequately measured by the EAT-16-Chronic Pain.

The prevalence of eating disorders identified in this sample of adolescents with chronic pain is considerably higher than that in the general population of adolescents (19.1 vs. 6.1%) [30]. Given that comorbid eating disorders involving active restrictive eating and/or purging behaviors can significantly interfere with program participation, participants with known eating disorders at the time of the pre-program evaluation are not deemed eligible for the progam until they receive successful treatment. As such, the prevalence of eating disorders identified in this sample may represent an underestimate of eating disorders in youth with chronic pain in the general population. Notably, the number of patients diagnosed with ARFID was also substantially higher than prevalence estimates from a large chart review study of pediatric gastroenterology patients (1.5%) [31], a finding which may be related to the severity of a patient population who requires intensive interdisciplinary pain treatment. Given that ARFID was the most common eating disorder in this sample and our findings validate that the EAT-26 is not a valid measure to screen for ARFID, it is important that ARFID specific screening measures such as the Nine Item ARFID Screen [32] be incorporated into the assessment of youth with chronic pain.

The findings of this study need to be considered within the context of several limitations. First, given the lack of socioeconomic and ethnic/racial diversity in this sample, it is unclear whether these results would generalize beyond a population of white, middle-class adolescents. Another clear limitation is that participants did not have their eating disorder confirmed with a structured clinical interview and only a subset of patients had an eating disorder evaluation. As such, it is possible this is an underestimate of eating disorders in this sample. However, all of the evaluations were conducted or supervised by a board-certified child and adolescent psychologist with a specialty in eating disorders, and referrals for eating disorder evaluations were based on parent and self-report of eating concerns, food logs, reviews of patient growth charts and behavioral observations over a three-week period, making it less likely that eating concerns were missed. Given that larger sample sizes are critical in factor analysis and add to the credibility of the results, a major strength of this study is the large mixed gender sample of adolescents with high impact chronic pain.

## Conclusions

In summary, factor analysis of the EAT-26 with adolescent patients with chronic pain yielded a five factor solution with scales highlighting fear of weight gain, concerns from others related to eating and weight, eating-related guilt, dietary restriction, and food preoccupation. The results suggest that the identified factor structure of the EAT-16-Chronic Pain is useful for identifying eating disorders other than ARFID in patients with high impact chronic pain and a new factor, *Social Pressure to Gain Weight* may assist in recognition of ARFID in conjunction with ARFID specific screening measures. Given that disordered eating seems to be a pertinent, yet often neglected area of inquiry in medical work up for chronic pain, this measure has potential to enhance the diagnosis and treatment of these adolescent patients.

## Data Availability

The datasets used and/or analysed during the current study are available from the corresponding author on reasonable request.
